# Vimentin as a contributing factor in SARS-CoV-2-induced orchitis on postmortem testicular autopsy of COVID-19 cases: A case-control study

**DOI:** 10.18502/ijrm.v22i11.17822

**Published:** 2025-01-10

**Authors:** Soheila Akaberi-Nasrabadi, Azam Sabbaghi, Behzad M. Toosi, Parsa Ghorbanifaraz, Gholam-Reza Mahmoudiasl, Abbas Aliaghaei, Hajarsadat Faghihi Hosseinabadi, Shahrokh Paktinat, Mohammad-Amin Abdollahifar

**Affiliations:** ^1^Urology and Nephrology Research Center, Research Institute for Urology and Nephrology, Shahid Beheshti University of Medical Sciences (SBMU), Tehran, Iran.; ^2^Department of Small Animal Clinical Sciences, University of Saskatchewan, Saskatoon, Saskatchewan, Canada.; ^3^Forensic Diagnostic and Laboratory Center of Tehran Province, Kahrizak, Tehran, Iran.; ^4^Department of Biology and Anatomical Sciences, School of Medicine, Shahid Beheshti University of Medical Sciences, Tehran, Iran.

**Keywords:** Autopsy, COVID-19, Orchitis, SARS-CoV-2, Vimentin.

## Abstract

**Background:**

Coronavirus disease 2019 (COVID-19) was identified in China in late December 2019 and led to a pandemic that resulted in millions of confirmed cases and deaths. The causative agent, severe acute respiratory syndrome coronavirus 2 (SARS-CoV-2), uses distinct receptors and co-receptors to enter host cells. Vimentin has emerged as a potential co-receptor for SARS-CoV-2 due to the high level of vimentin expression in testis tissue.

**Objective:**

The present study investigated the link between vimentin expression level and SARS-CoV-2-induced orchitis.

**Materials and Methods:**

In this case-control study, testis autopsy samples were collected immediately after the death of both COVID-19 cases and a control group that included individuals who died due to accidental causes. Gene expression and immunohistochemical assays were conducted to evaluate the level of vimentin expression, cell proliferation, and leukocyte infiltration.

**Results:**

A significant expression of vimentin and infiltration of immune cells (CD68+, CD38+, and CD138+) in the testicular tissue of COVID-19 cases, along with extensive immunoglobulin G precipitation and reduced inhibin expression (p = 0.001) were observed. Additionally, gene expression analysis revealed increased expression of vimentin and decreased expression of the proliferation markers Ki67 and proliferating cell nuclear antigen, suggesting that SARS-CoV-2 may disrupt spermatogenesis through immune responses and the arrest of cell proliferation.

**Conclusion:**

There may be a strong link between vimentin expression and COVID-19-induced orchitis. Further studies are needed to confirm these findings. Considering some limitations, vimentin can be used as a biomarker option for testicular damage following COVID-19-induced orchitis.

## 1. Introduction

The COVID-19 pandemic, triggered by the severe acute respiratory syndrome coronavirus 2 (SARS-CoV-2), was declared in March 2020, shortly after its first appearance in Wuhan, China, in December 2019. Since then, millions of confirmed cases and deaths have been reported worldwide (https://covid19.who.int/), and it remains a persistent cause of symptoms despite widespread vaccination efforts. Multiorgan involvement is a significant manifestation of COVID-19 with the male reproductive system among the affected organs (1). As a result, there is still concern regarding the effects of COVID-19 on male fertility. The histopathology of COVID-19 in testis tissue has been studied during the pandemic; however, multiple questions remain, including the tropism of SARS-CoV-2 toward testis tissue and its ability to induce direct orchitis (2, 3).

Angiotensin-converting enzyme II (*ACE2*) acts as the main receptor, enabling SARS-CoV-2 entry into host cells. However, the interaction between the SARS-CoV-2 spike protein and *ACE2* is insufficient for viral entry. When a viral particle binds to *ACE2*, entrance is primed by the function of transmembrane protease serine 2 (*TMPRSS2*), which is found on the host-cell surface (4). Therefore, the presence of both *ACE2* and *TMPRSS22* is critical for SARS-CoV-2 entry, as confirmed by studies showing a considerable level of *ACE2* and *TMPRSS2* co-expression in affected organs. Since a low level of *ACE2* and *TMPRSS2* co-expression exists in testicular cells, a reduced ability of SARS-CoV-2 entry into testicular cells and consequent decreased likelihood of its direct pathogenesis in testis tissue would be expected (5, 6). However, ultrastructural analysis revealed the presence of SARS-CoV-2 viral particles in testis tissue and suggested a link between viral presence and infiltration of leukocytes into the testis, causing orchitis (7).

Vimentin, an intermediate filament of the cytoskeleton, has been demonstrated to be involved in the process of fusion and entry of severe SARS-CoV. Indeed, a direct interaction between the virus spike protein and surface vimentin was detected, making vimentin a potential co-receptor involved in SARS-CoV entry (8). In addition, there are reports highlighting the involvement of vimentin during viral replication and assembly (9). In the testis tissue, vimentin is expressed by multiple cells, including Sertoli cells and different populations of interstitial cells such as Leydig cells, peritubular myoid cells, as well as endothelial cells, fibroblasts, and macrophages (10, 11). Due to the high level of vimentin expression in Sertoli cells, it is also considered a marker of these cells (11). Based on the evidence of vimentin's direct interaction with the SARS-CoV spike protein throughout the spike-ACE2 attachment process (8), we postulated that high expression of vimentin might be a susceptibility factor of testis tissue in the severely affected cases who died of COVID-19.

To further understand the effects of SARS-CoV-2 on testicular tissue, we also examined the expression of a proliferation marker Ki67 and proliferating cell nuclear antigen (*PCNA*) genes. Ki67 and PCNA are well-established markers of cell proliferation and are crucial for assessing the impact of injuries on testicular tissue (12). By analyzing these markers, we aimed to determine whether SARS-CoV-2 infection leads to alterations in cell proliferation, which could contribute to spermatogenesis arrest and other testicular pathologies.

Therefore, this study was conducted to evaluate if there is an effect of SARS-CoV-2 on the testis tissue of deceased cases by investigating histopathological changes and evaluating if a relationship between vimentin overexpression and the degree of damage caused by direct damage orchitis is observed. To characterize the cases of orchitis, we also studied the degree of leukocyte infiltration and immunoglobulin G (IgG) deposition in the testis tissue of COVID-19 cases.

## 2. Materials and Methods

### Patient selection and study design

This case-control study aimed to compare pathological features of testicular tissue between postmortem COVID-19 cases and a control group. 20 testicular specimens were collected from the Forensic Medicine Organization of Tehran, Kahrizak Center, Tehran, Iran between April 15, 2022 and the end of June 2023. 10 samples were from men who died due to COVID-19, and 10 from individuals who died from accidental causes (e.g., trauma, electrocution, or carbon monoxide poisoning), all of whom had no comorbidities and tested negative for COVID-19.

Inclusion criteria required adult males with no history of reproductive disorders or infertility. For the COVID-19 group, SARS-CoV-2 positivity was confirmed via reverse transcription polymerase chain reaction (RT-PCR) and clinical findings. The control group included individuals with proven fertility and no reproductive health issues. Exclusion criteria applied to participants with a history of reproductive disorders, significant genital pathology, or infertility in both groups.

Samples were collected 8–10 hr postmortem, ensuring minimal tissue degradation. Testicular samples, each measuring approximately 3 × 3 cm², were obtained via autopsy from different regions of the testis. Bodies were then refrigerated to preserve tissue integrity, and autopsies were conducted promptly at the Forensic Medicine Organization of Tehran, Kahrizak Center, Tehran, Iran. Each sample was obtained for histopathological and molecular analysis. Written informed consent was obtained from family members. The study followed strict legal and ethical guidelines, and testicular samples were handled consistently to ensure comparability between the groups.

### Sample size

The sample size of 10 cases and 10 controls was determined based on previous studies and practical considerations, including postmortem sample availability. Power analysis was conducted, accounting for an α level of 0.05 and power ( β ) of 0.20 to ensure robustness. A slightly larger sample size was included to account for potential sample loss. Details of the power analysis formula are provided in the supplementary material.

### Study design and setting

COVID-19 cases were confirmed through molecular diagnostics, while controls, with proven fertility and no reproductive disorders, were selected based on medical history and autopsy reports. Data on relevant variables such as age, comorbidities, and corticosteroid therapy were recorded for both groups.

Variables included: 1) outcomes: vimentin expression, cell proliferation, leukocyte infiltration; 2) exposures: COVID-19 status; 3) confounders: age, cause of death, comorbid health conditions.

Samples were processed using standardized histopathological and molecular methods, ensuring consistency across the study.

### Tissue preparation and staining

Autopsy samples for gene expression study were collected in RNAlater solution and immediately transferred to the laboratory. Samples for the stereology study were collected in Bouin's solution, kept for 48 hr, and then transferred to 10% buffered formalin for an extra 24 hr. Afterward, fixed tissues were processed, embedded in paraffin, and cut into thin sections using a microtome (Leica, Germany). Sections of testis tissues were taken serially at 25 µm to estimate the number of defined cells. Then, 10 sections of each testis sample were selected using the systematic uniform random sampling method, which was done by picking a random section in the 1–10 range.

Next, the sections were stained using hematoxylin and eosin for histopathology and stereology evaluations. Finally, 2 expert histologists analyzed the tissue sections based on a blind review of the data analysis in the COVID-19 group vs. control group. In this study, for positive controls, samples containing the target molecule at its known location (e.g., adrenocortical adenoma, leiomyosarcoma, brain tumor, tonsil, bone marrow) were used.

### Immunohistochemical staining

For immunohistochemical staining, mouse antihuman vimentin and mouse antihuman inhibin alpha (Vitro, Spain), as markers of Sertoli and Leydig cells; mouse antihuman CD68 (Vitro, Spain), as a marker of macrophages; polyclonal rabbit antihuman IgG (Dako, Denmark); mouse antihuman CD38 and mouse antihuman CD138 (GenomeMe, Canada), as markers of B cells and plasma cells were used. Paraffin sections of the testis tissue were first deparaffinized using xylene. Then, the sections were hydrated in a series of decreasing concentrations of alcohol solution and rinsed in phosphate-buffered saline (PBS) (Biowest, France). Afterward, they were boiled for 15 min in 10 mM citrate buffer in a microwave (700 watts) for epitope retrieval. After cooling the sections were incubated at room temperature for 15 min, then washed 3 times with PBS. They were subsequently treated with 5% bovine serum albumin at room temperature for 60 min. Following this, the sections were incubated with primary antibodies. Diluted 1 in 100 with PBS containing Tween-20, overnight at 4 C. After applying appropriate secondary antibodies (diluted 1 in 200) (Dako, Denmark), sections were incubated with 3'-diaminobenzidine 3,3'-diaminobenzidine (Dako, Denmark). After a final wash in PBS, sections were eventually dehydrated, fixed, counterstained with hematoxylin (Merck, USA), mounted and examined under a light microscope (Nikon, Japan). To quantify the intensity and immunoreactivity of protein expression, we measured the optical density using the following formula: optical density = log (max intensity/mean intensity), where the maximum intensity is set to 255 for 8-bit images.

### Analysis of *vimentin*, *Ki67*, and *PCNA* gene expression

To evaluate the gene expression levels of vimentin, Ki67 (a marker of cell proliferation), and PCNA (another marker of cell proliferation), quantitative RT-PCR was performed. First, total RNA was extracted from the samples using a commercial kit (GeneAll, South Korea) and treated with DNase I (Roche, Basel, Switzerland) to remove any genomic DNA contamination. cDNA synthesis was performed using a commercial kit (Fermentas, Lithuania) according to the protocol described in the manufacturer's instructions. Quantitative RT-PCR was conducted using a QuantiTect SYBR Green RT-PCR kit (QIAGEN, Germany), which was used to quantify the relative gene expression according to the kit's instructions. We confirmed the specificity of the primer sets (forward and reverse) by using the NCBI BLAST tool (http://www.ncbi.nlm.nih.gov/BLAST), aligning them with sequences from the NCBI database (Table I). The *GAPDH* gene was used as a housekeeping gene.

**Table 1:** Primers' design

**Gene name**	**Primers sequences**
* * ***GAPDH***	F: AAAGAGATAATCTGGCTCTGC R: GCTCTGAGACAATGAACGCT
* * ***Ki67***	F: ATTCCGACTGTCCAGAAGCA R: GCTGGATGTGATGGCTGATG
* * ***PCNA***	F: GTACACGCCGCTGTATTCG R: GAGATCCTTGAGTGCCTCCA
* * ***Vimentin***	F: AGTCCACTGAGTACCGGAGAC R: CATTTCACGCATCTGGCGTTC
*GAPDH*: Glyceraldehyde 3-phosphate dehydrogenase, *Ki67*: Marker of proliferation Ki67, *PCNA*: Proliferating cell nuclear antigen, F: Forward, R: Reverse	

### Ethical Considerations 

This study was approved by the Ethics Committee of Shahid Beheshti University of Medical Sciences, Tehran, Iran (Code: IR.SBMU.UNRC.REC.1401.006). Written informed consent was obtained from the families of all deceased cases prior to the postmortem examination.

### Statistical Analysis

Independent sample data were analyzed using Statistical Package for the Social Sciences, version 23.0, SPSS Inc., Chicago, Illinois, USA. The normal distribution of quantitative data was analyzed using Kolmogorov-Smirnov test. Unpaired Student's *t* tests was used for statistical analysis. For gene expression analysis, RT-qPCR data were processed using the 2 ${\;}^{-\Delta \Delta \text{CT}}$ method. Statistical significance was considered at a p-value ≤ 0.05. Data are presented as mean ± standard deviation (SD).

## 3. Results

### Participantdemographics and clinical characteristics

The age distribution of participants in both the COVID-19 and control groups was similar, with a mean age of 53 ± 4 yr. The duration of COVID-19 illness, among cases, ranged from 20–31 days. None of the participants in either group had a history of reproductive system disorders. All individuals, both in the COVID-19 and control groups were fertile and had children. All COVID-19 cases received corticosteroid therapy. Comorbidities observed among the COVID-19 cases included hypertension and type 2 diabetes. The causes of death for COVID-19 cases included pneumonia, acute respiratory distress syndrome, respiratory failure, septic shock, renal failure, and myocardial infarction (Table II, and III).

### Expression of vimentin, inhibin, CD68, IgG, CD38, and CD138 in the testis of COVID-19 cases

**Figure 1:**
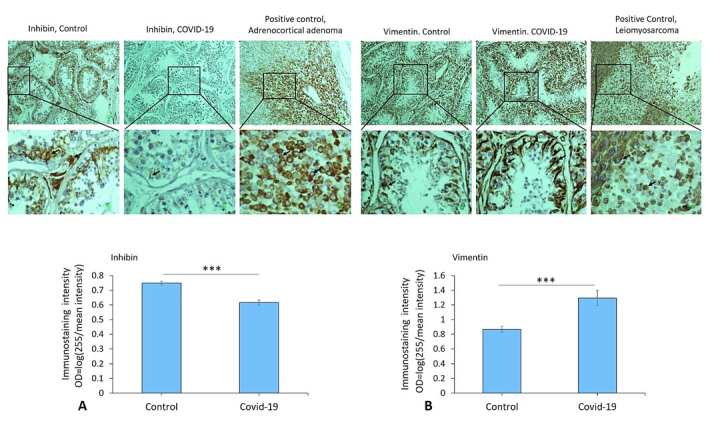
A) The immunostaining patterns of vimentin and inhibin in control and COVID-19 groups. Immunoreactivity pattern (OD = log [max intensity/mean intensity]) of vimentin and inhibin in the testis in study groups. B) The significant difference between COVID-19 and control groups is indicated (***P < 0.001). Arrows indicate vimentin+ and inhibin+ cells. An unpaired two-tailed *t* test has been used for the comparisons.

**Figure 2:**
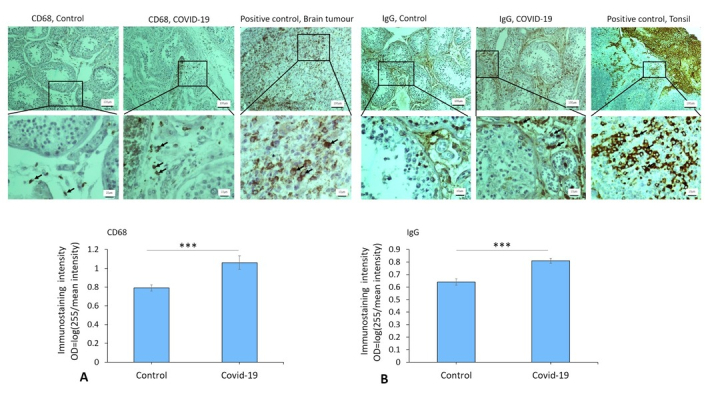
A) The immunostaining patterns of CD68 and IgG in control and COVID-19 groups. Immunoreactivity pattern (OD = log [max intensity/mean intensity]) of CD68 and IgG in testis in study groups. B) The significant difference between COVID-19 and control groups is indicated (***P < 0.001). Arrows indicate CD68+ and IgG+ cells. An unpaired two-tailed *t* test has been used for the comparisons.

**Figure 3:**
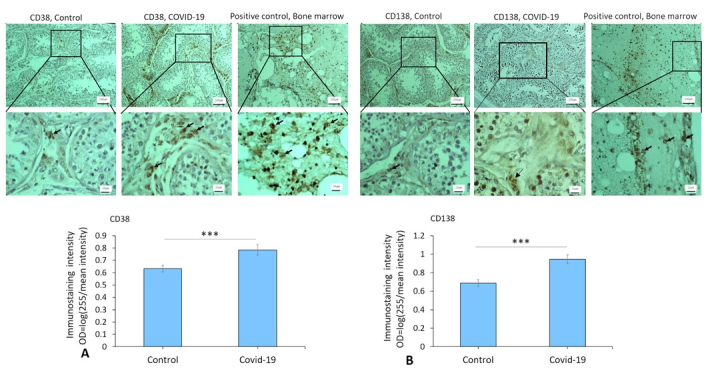
A) The immunostaining patterns of CD38 and CD138 in control and COVID-19 groups. Immunoreactivity pattern (OD = log ([max intensity/mean intensity]) of CD38 and CD138 in testis in study groups. B) The significant difference between COVID-19 and control groups is indicated (***P < 0.001). Arrows indicate CD38+ and CD138+ cells. An unpaired two-tailed *t* test has been used for the comparisons.

Immunostaining data demonstrated an increased expression of vimentin (as a potential co-receptor for SARS-CoV-2) in the COVID-19 group in comparison to controls (p < 0.001) (Figure 1). In addition, immunohistochemical staining revealed severe infiltration of CD68+ (macrophages), CD38+ (activated B cells), and CD138+ (plasma cells) in the interstitial compartments of testis tissue of the COVID-19 group. In contrast, such infiltration was rarely detected in controls (Figure 2, and 3). Correspondingly, extensive IgG precipitation was observed mainly in the germinal epithelium of seminiferous tubules and interstitial tissue of the testis in the COVID-19 group (Figure 2). Meanwhile, the expression of inhibin, as a marker of Sertoli and Leydig cells, was significantly reduced in the COVID-19 group compared to controls (p < 0.001). These data suggest that SARS-CoV-2 might enter testis tissue using vimentin as its co-receptor and trigger a secondary autoimmune response contributing to the primary pathogenesis of viral orchitis, consequent testicular damage, and reduced number of testis functional cells.

### Expression of *vimentin*, *Ki67*, and *PCNA* genes in the testis of COVID-19 cases

**Figure 4:**
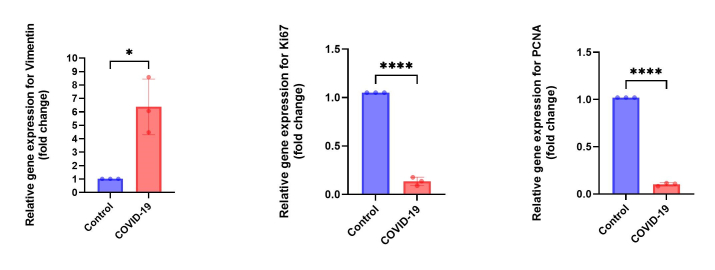
mRNA expression level of* vimentin* (*P = 0.0110), *Ki67* (****P < 0.0001), and *PCNA *(****P < 0.0001) in the testis. An unpaired two-tailed *t* test.

Similar to data from immunostaining, the gene expression level of *vimentin* was significantly upregulated in the COVID-19 group in comparison to the control group (p < 0.05). Meanwhile, the transcripts for genes involved in cell proliferation, such as *Ki67* and *PCNA*, were significantly downregulated in the COVID-19 group as compared to the control group (p < 0.0001) (Figure 4). These results imply decreased cell proliferation resulting from SARS-CoV-2-induced spermatogenesis arrest.

**Table 2:** Clinical characteristics of COVID-19 cases

**Case no.**	**Age (yr)**	**Disease duration (days)**	**Corticosteroid therapy**	**Comorbidity**	**Cause of death** **(COVID-19)**
**Case 1**	57	30	Yes	Hypertension	Respiratory and cardiovascular conditions
**Case 2**	53	23	Yes	Type 2 diabetes	Lung failure and severe infection
**Case 3**	49	26	Yes	Non	Respiratory failure, septic shock
**Case 4**	50	27	Yes	Type 2 diabetes	Lung failure and severe infection
**Case 5**	57	30	Yes	Hypertension	Respiratory failure, myocardial infarction
**Case 6**	55	24	Yes	Hypertension, type 2 diabetes	Respiratory and cardiovascular conditions
**Case 7**	50	20	Yes	Hypertension	Lung failure, renal failure and severe infection
**Case 8**	54	29	Yes	Non	Renal failure, respiratory and cardiovascular conditions
**Case 9**	57	31	Yes	Non	Respiratory and cardiovascular conditions and severe infection
**Case 10**	49	27	Yes	Hypertension	Respiratory and cardiovascular failure
None of the participants had reproductive system disorder and all were fertile with a child					

**Table 3:** Clinical characteristics of the control group

**Case no.**	**Age (yr)**	**Cause of death (accidental causes)**
**Case 1**	55	Collision
**Case 2**	50	Electrocution
**Case 3**	49	Carbon monoxide poisoning
**Case 4**	54	Collision
**Case 5**	49	Trauma
**Case 6**	56	Collision
**Case 7**	48	Trauma
**Case 8**	48	Collision
**Case 9**	51	Trauma
None of the participants had reproductive system disorder and all were fertile with a child		

## 4. Discussion

The present study showed that vimentin expression was significantly high in the testis tissues of cases who died of severe COVID-19. Simultaneously, the level of leukocyte (i.e., macrophages and plasma cells) infiltration, IgG deposition, and tissue destruction were notably high in SARS-CoV-2-infected testes. The remarkable manifestation of injury was the reduced number of all types of germ cells, Sertoli and Leydig cells, a substantial decrease in the expression of inhibin as a marker of Sertoli and Leydig cells, and a significant reduction in cell proliferation markers, that is,* Ki67* and *PCNA*. Due to the possibility of vimentin involvement during SARS-CoV-2 invasion into testicular tissue cells (13) and its role in inflammation and immune response (14, 15), these data suggest that men with higher levels of vimentin in the testis tissue, which are infected with COVID-19 virus, may be more sensitive to orchitis. These findings may provide insight into the mechanism of SARS-CoV-2 entry into testicular cells and its subsequent pathogenesis and unveil a possible target for therapeutic intervention against the impact of COVID-19 on male fertility.

Previous studies suggested that the high viral load of SARS-CoV-2 is linked to the severity of COVID-19 infection (16). The co-expression of ACE2 and TMPRSS2 as main participants during SARS-CoV-2 entry has been reported to be very low in testicular cells according to data obtained from single-cell RNA sequencing analysis. These data suggested a low probability of severe and long-term impacts of COVID-19 infection on male gonads (6). However, reports of SARS-CoV-2 presence in testis tissue (7) and multiple studies showing moderate to severe testicular damage observed in autopsy samples from fatal COVID-19 cases (17, 18) strengthens the hypothesis of the testis being a target organ for SARS-CoV-2. In this regard, one of the most important questions unanswered is how SARS-CoV-2 enters testicular cells without using its common receptor and co-receptor.

Indeed, although ACE2 has been recognized as the essential receptor, not all ACE2-expressing cells are infected equally, suggesting a role for other factors in host-cell invasion by SARS-CoV-2. Vimentin is generally considered an intracellular protein; however, there is growing evidence demonstrating that it is also present in the extracellular space, especially on the cells surface (19, 20), where it can act as a receptor or co-receptor for different pathogens, including viruses and bacteria (21–23). Recently, vimentin has been identified as a strong candidate for being a co-receptor of SARS-CoV-2 (24), similar to what happens during the invasion of SARS-CoV as the prototype virus. The Spike protein of SARS-CoV, identical to that of SARS-CoV-2, is assumed to be the binding protein for vimentin located on the external surface of host cells (8). The potential interactions between the SARS-CoV-2 spike protein and superficial vimentin need further investigation to confirm this mechanism as an alternative route for SARS-CoV-2 virion entry in tissues like testis that lack significant co-expression of ACE2 and TMPRSS2; however, have significant expression of vimentin in its different functional cell types, including Sertoli cells and Leydig cells.

Vimentin is also a critical participant during the immune response because it promotes inflammatory reactions and is required for immune cell signaling (15, 25). Vimentin has been described as a ligand for some pattern recognition receptors or innate immune receptors (26). In addition, leukocyte extravasation has been connected to vimentin (27). Previous studies have demonstrated a rapid increase in vimentin expression in response to inflammatory stimuli consequent to viral infection (25). Vimentin is part of a category of genes known as immediate early genes, which are activated soon after infection and inflammation occurs (13, 25). Based on these data, vimentin overexpression in the testis tissue of COVID-19 cases, as observed in the present study, may also play a role during the uncontrolled immune response, a characteristic of SARS-CoV-2 infection. The induction of both cellular and humoral immune reactions is reported in COVID-19 cases. Likewise, different studies have demonstrated the infiltration of various immune cells, including macrophages, T and B lymphocytes, and plasma cells, into testis tissue in COVID-19 cases (28). Similar results were observed in the present study, showing that the number of CD68+ macrophages and CD38+ CD138+ activated B cell/plasma cells were higher than control cases, who were negative for the SARS-CoV-2 genome at the time of death. In parallel to the presence of humoral immune cells, widespread precipitation of IgG was shown in different compartments of SARS-CoV-2-infected testes (17, 18). In line with these data, we revealed that the level of IgG was very high in the testis tissue of COVID-19 cases. The findings indicate that the humoral immune-mediated reaction is a secondary immune response contributing to the initial pathogenesis of viral orchitis in COVID-19 cases. This reaction leads to the degeneration of testicular cells in both the germinal epithelium and interstitial cells, as well as a reduction in Sertoli cells' product, specifically inhibin. This condition is similar to autoimmune orchitis, which was previously detected in SARS-CoV-infected cases (29). Collectively, this excess immune response is now considered to be the main contributor to the pathogenesis of SARS-CoV-2 in male gonads, causing spermatogenic dysfunction (14).

Our findings highlight a marked decrease in cell proliferation within the testes, which correlates with the observed reduction in germ cells, Sertoli cells, and Leydig cells. Ki67 and PCNA are critical indicators of cell proliferation, and their reduced expression suggests impaired cellular regeneration and compromised tissue homeostasis in the context of viral orchitis induced by SARS-CoV-2. The decreased proliferation may contribute to the overall testicular damage, as reflected by the lower numbers of functional testicular cells and the elevated levels of inflammatory markers. These results support the hypothesis that SARS-CoV-2 infection not only causes direct damage to testicular tissues but also disrupts normal regenerative processes, which may have long-term implications for male fertility.

Taken together, the existing evidence suggests a link between vimentin expression and COVID-19-induced orchitis. Data regarding the pathological impacts of the infection on male gonads are strong enough to encourage scientists to investigate the mechanisms by which vimentin is involved in SARS-CoV-2 pathogenesis in the testis. Finding the SARS-CoV-2 target cell in the testis may aid in ending the debate about the presence or absence of the virion in semen, the possibility of sexual transmissibility, and the effects on sperm quality. Therefore, further research should focus on finding the target testicular cells for the virus, the mechanism of virion entry using vimentin as a potential uncommon co-receptor, the participation of vimentin during viral replication and assembly like other viral infections, and the contribution of vimentin to the infiltration of immune cells into the testis and subsequent inflammation and possible autoimmune reactions.

## 5. Conclusion

These data suggest that there may be a strong link between vimentin expression and COVID-19-induced orchitis. Further studies are required to confirm our preliminary findings by determining the relationship between the severity of testis tissue damage, viral load in the tissue, and expression level of vimentin in a large-scale setting. Further elucidation of the role that vimentin performs in mediating SARS-CoV-2 pathogenesis may identify vimentin as a valuable target for therapeutic intervention at the early stages of COVID-19 infection to avoid the adverse effects of SARS-CoV-2 on male reproductive function. Significant limitations of the present study include a small sample size and a lack of investigation for viral detection in testis tissue.

##  Data Availability

The data and materials supporting this study's findings are available from the corresponding author upon reasonable request.

##  Author Contributions

MA. Abdollahifar and Sh. Paktinat designed this study and conducted the stereological study, molecular test, and drafted the manuscript. S. Akaberi-Nasrabadi and A. Sabbaghi carried out the immunohistochemistry, histological technique, and provided the clinical data. BM. Toosi and A. Aliaghaei helped to draft the manuscript and carried out the molecular test. P. Ghorbanifaraz helped to draft the manuscript. H. Faghihi Hosseinabadi performed the statistical analysis. GhR. Mahmoudiasl provided the clinical sample. All authors read and approved the final manuscript.

To ensure fairness and accurately reflect contributions, this article designates 2 first authors, Soheila Akaberi-Nasrabadi and Azam Sabbaghi, as they both equally contributed to the experimental work, including immunohistochemistry, histological techniques, and clinical data collection, which form the core of the project. Similarly, we have 2 corresponding authors, Shahrokh Paktinat and Mohammad-Amin Abdollahifar, who jointly designed the study, conducted the stereological analysis, performed real-time PCR, and contributed to drafting the manuscript. This dual authorship structure for both first and corresponding authors is essential to acknowledge the balanced and collaborative efforts that were central to this study's success.

##  Acknowledgments 

The present article is supported by “Urogenital Stem Cell Research Center, Shahid Beheshti University of Medical Sciences, Tehran, Iran” (Registration No. 31054). This work was conducted in the Department of Biology and Anatomical Sciences, School of Medicine, Shahid Beheshti University of Medical Sciences, Tehran, Iran. Special thanks are extended to the Forensic Center of Tehran, Iran, for their collaboration. Additionally, we used artificial intelligence (Wordtune) to a limited extent to paraphrase some sentences in this manuscript, including some specific sentences in the methods section and statistical analysis section.

##  Conflict of Interest

The authors declare that they have no competing interest.
